# Bletilla striata oligosaccharides alleviate high-fat diet-induced metabolic associated fatty liver in mice through modulation of gut microbiota and host metabolism

**DOI:** 10.3389/fnut.2026.1852189

**Published:** 2026-07-08

**Authors:** Kaiwen Lei, Jie Li, Kunhua Wei, Yan Bai, Jianwen Mao, Zhengquan Su

**Affiliations:** 1 Guangdong Engineering Research Center of Natural Products and New Drugs, Guangdong Provincial University Engineering Technology Research Center of Natural Products and Drugs, Guangdong Pharmaceutical University, Guangzhou, China; 2 Guangdong Provincial Key Laboratory of Pharmaceutical Bioactive Substances and School of Basic Medical Sciences, Guangdong Pharmaceutical University, Guangzhou, China; 3 Key Laboratory of State Administration of Traditional Chinese Medicine for Production and Development of Cantonese Medicinal Materials, Guangzhou Comprehensive Experimental Station of National Industrial Technology System for Chinese Materia Medica, Guangdong Engineering Research Center of Good Agricultural Practice and Comprehensive Development for Cantonese Medicinal Materials, School of Chinese Materia Medica, Guangdong Pharmaceutical University, Guangzhou, China; 4 Health Sciences Academy, Guangzhou Nanyang Polytechnic College, Guangzhou, China

**Keywords:** Bletilla striata oligosaccharides, gut-liver axis, hepatic inflammatory response, lipid metabolism, metabolic associated fatty liver disease

## Abstract

**Background and aim:**

Gut-liver axis dysfunction drives metabolic associated fatty liver disease (MAFLD), but effective therapeutic strategies remain limited. Bletilla striata oligosaccharides (BSO) have immunomodulatory potential, yet their role in MAFLD via the gut-liver axis is unclear. This study aimed to investigate whether and how BSO ameliorates MAFLD by modulating gut microbiota, intestinal barrier function, and hepatic inflammation.

**Results:**

MAFLD was induced in mice by 8-week high-fat diet followed by 12-week BSO (150, 300, 600 mg/kg) or metformin treatment via oral gavage. Compared with the MAFLD model, high-dose BSO reduced body weight gain, lowered fasting glucose, and decreased hepatic triglycerides. BSO also attenuated liver injury, hepatic steatosis, inflammation. Mechanistically, BSO restored gut barrier integrity, upregulated colonic tight junction proteins, activated colonic LXRα/ABCA1 signaling, while suppressing the hepatic TLR4/NF-κB pathway. BSO remodeled gut microbiota, enriching beneficial Lachnospiraceae and Oscillospiraceae, and modulated hepatic metabolites, as shown by decreased confertifoline along with increased D-myo-inositol-4-phosphate. Additionally, BSO activated the intestinal FXR/FGF15 axis and ameliorated bile acid metabolism disorders, evidenced by reduced tauro-ω-muricholic acid and cholic acid.

**Conclusion:**

This study provides systematic evidence that BSO alleviates MAFLD through a multi-target gut-liver axis mechanism involving gut microbiota remodeling, barrier restoration, activation of LXRα/ABCA1 and FXR/FGF15 signaling, and subsequent suppression of hepatic TLR4/NF-κB-driven inflammation. Compared to previous approaches, BSO offers a favorable safety profile with combined regulatory effects. These findings support BSO as a promising candidate for MAFLD treatment, with potential applications as a dietary supplement or prebiotic agent.

## Graphical Abstract

**Figure:**
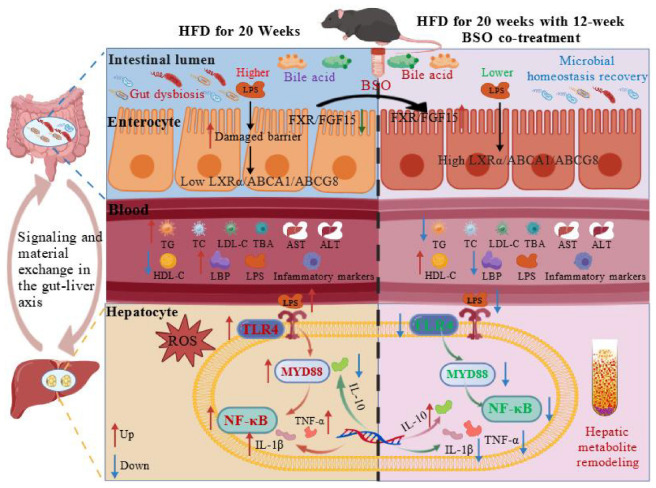
Bletilla striata oligosaccharides regulate gut microbiota and host metabolism via the gut-liver axis to alleviate metabolic associated fatty liver disease. Created with BioGDP.com.

## 1 Introduction

Metabolic associated fatty liver disease (MAFLD) is a metabolic disorder characterized by hepatic steatosis as its primary pathological feature. Its diagnosis requires the presence of hepatic steatosis confirmed by imaging or histology, along with at least one metabolic abnormality such as overweight/obesity, type 2 diabetes mellitus, or metabolic syndrome (1, 2). Since the international panel of liver disease experts proposed the new nomenclature of MAFLD in 2020, this term has rapidly replaced the traditional concept of non-alcoholic fatty liver disease (NAFLD), more accurately reflecting its intrinsic association with metabolic dysregulation (3). Epidemiological surveys indicate that the global prevalence of MAFLD has increased from 25.26% during 1990–2006 to 38.00% during 2016–2019, with the highest prevalence reaching 44.37% in Latin America and 29.71% in East Asia (4). The disease burden is further compounded by the emergence of lean MAFLD, a distinct phenotype particularly prevalent in Asian populations, accounting for approximately 5.1% of the global population and up to one-third of MAFLD cases in Asia, yet frequently underdiagnosed due to the absence of overt obesity (4, 5). More concerningly, MAFLD not only can progress to cirrhosis and hepatocellular carcinoma but also independently increases the risk of type 2 diabetes mellitus, cardiovascular disease, and chronic kidney disease (6, 7). However, currently only Resmetirom, a thyroid hormone receptor β agonist, has been approved globally for the treatment of metabolic associated steatohepatitis (MASH) with liver fibrosis, leaving therapeutic options for MAFLD still very limited (8). This stark treatment gap, coupled with the rising global epidemic of obesity and metabolic syndrome, underscores the urgent necessity for developing effective and accessible interventions for MAFLD.

The pathogenesis of MAFLD is complex. According to the classic multiple-hit theory, on the basis of insulin resistance and lipid metabolism disorders, multiple factors including oxidative stress, endoplasmic reticulum stress, inflammatory responses, and gut microbiota dysbiosis collectively drive the onset and progression of the disease (9, 10). Among these, the gut-liver axis, as a bidirectional communication pathway connecting the intestine and the liver, plays a critical role in the pathogenesis of MAFLD (11). In this context, assessing metabolic conditions and nutritional parameters affecting gut microbiota is of paramount importance. Dietary fiber has been shown to modulate gut microbiota composition and metabolic functions in obese models (12). As the body largest reservoir of microorganisms and a major immune organ, the integrity of the intestinal barrier function is essential for maintaining liver homeostasis. Long-term high-fat diet (HFD) can lead to gut microbiota dysbiosis, characterized by an increased Firmicutes/Bacteroidetes ratio and a reduction in beneficial bacteria such as Bifidobacterium (13, 14). Multi-omics studies have revealed characteristic microbial signatures in MAFLD, including depletion of butyrate-producing genera Faecalibacterium and Coprococcus, alongside expansion of pro-inflammatory genera such as Escherichia and Dorea (15). This dysbiosis disrupts the integrity of intestinal tight junctions, leading to increased intestinal permeability and the translocation of bacterial products, such as lipopolysaccharide (LPS), to the liver via the portal vein (16). LPS binds to Toll-like receptor 4 (TLR4) on the surface of hepatic macrophages, activating nuclear factor κB (NF-κB) through a myeloid differentiation factor 88 (MYD88)-dependent pathway, which in turn initiates the transcription of downstream pro-inflammatory cytokines such as tumor necrosis factor-α (TNF-α) and interleukin-1β (IL-1β), driving chronic hepatic inflammation (17, 18). Therefore, restoring the intestinal barrier and blocking the LPS-TLR4 inflammatory axis are considered important strategies for intervening in MAFLD.

In recent years, the role of gut-derived high-density lipoprotein (HDL) in regulating hepatic inflammation has garnered significant attention. HDL not only participates in reverse cholesterol transport but also possesses potent LPS-neutralizing capacity (19). Liver X receptor α (LXRα) is a key nuclear receptor regulating HDL synthesis; its activation upregulates the expression of ATP-binding cassette transporter A1 (ABCA1) and ABCG8 in intestinal epithelial cells, promoting the efflux of cholesterol and phospholipids to apolipoprotein A1 (ApoA1) and the formation of nascent HDL (20, 21). More importantly, recent studies have found that gut-synthesized HDL can bind to LPS in the portal circulation, preventing LPS from binding to TLR4 on hepatocytes, thereby exerting anti-inflammatory effects (22–24). Notably, LPS can directly interfere with reverse cholesterol transport by reducing LXR expression, establishing a vicious cycle wherein gut dysbiosis promotes inflammation while simultaneously impairing the host endogenous anti-inflammatory HDL production (25). This suggests that the intestinal LXRα/ABCA1 pathway may serve as a critical node linking intestinal lipid metabolism to hepatic inflammation by regulating the production of gut-derived HDL (25). However, whether natural products can therapeutically augment this gut-derived HDL pathway to alleviate MAFLD remains a critical yet unexplored question.

Furthermore, bile acids, as another class of important signaling molecules in the gut-liver axis, participate in the regulation of MAFLD by activating the farnesoid X receptor (FXR) (26). Primary bile acids are synthesized in the liver and excreted into the intestine with bile, where they are converted into secondary bile acids by the gut microbiota. Bile acids are endogenous ligands of FXR. Upon activation in the intestine, FXR induces the expression and secretion of fibroblast growth factor 15 (FGF15). FGF15 returns to the liver via the portal vein and inhibits the expression of cholesterol 7α-hydroxylase (CYP7A1), exerting negative feedback regulation of bile acid synthesis (27). Clinical studies have shown that patients with MAFLD exhibit significant bile acid metabolic disorders, including elevated total bile acid levels, altered proportions of secondary bile acids, and impaired FXR signaling (28, 29). Restoring the normal function of the FXR/FGF15 signaling axis can ameliorate hepatic steatosis, inflammation, and fibrosis (30). The convergence of bile acid signaling with gut microbiome composition further highlights the importance of assessing these nutritional and metabolic parameters in MAFLD (5, 27). Comprehensive reviews have emphasized that dietary fiber and polysaccharides can remodel the gut microbiota and restore bile acid homeostasis, providing a strong mechanistic rationale for plant-derived interventions (31).

Bletilla striata (Thunb.) Reichb. f. is a plant belonging to the Orchidaceae family, and its dried tuber is used as a traditional Chinese medicine. It is traditionally used for its astringent, hemostatic, detumescent, and tissue-regenerating properties. Modern pharmacological studies have demonstrated that Bletilla striata polysaccharides possess antioxidant, anti-inflammatory, immunomodulatory, and hepatoprotective effects (32, 33). Dietary intervention with Bletilla striata polysaccharides has been shown to reshape the hepatic lipid profile and alleviate intestinal metabolic disorders in high-fat diet-induced obese mice (34). Bletilla striata oligosaccharides (BSO) are degradation products of Bletilla striata polysaccharides, characterized by low molecular weight, good water solubility, and high bioavailability. Previous studies have shown that BSO ameliorates glucose and lipid metabolic disorders in HFD-induced obese mice and modulates gut microbiota structure (35, 36). Additionally, in a DSS-induced ulcerative colitis model, BSO has been shown to suppress inflammatory responses by regulating the NLRP3 inflammasome (37). Despite these promising findings, the precise mechanisms by which BSO improves MAFLD remain incompletely understood. Specifically, it remains unknown whether BSO exerts hepatoprotective effects through the intestinal LXRα/ABCA1-HDL-LPS pathway, a critical link between gut dysbiosis and hepatic inflammation. Additionally, whether BSO modulates the FXR/FGF15 bile acid signaling pathway to restore bile acid homeostasis has not been explored.

To address these unanswered questions, the present study aimed to investigate the mechanisms of action of BSO in MAFLD. We employed an HFD-induced MAFLD mouse model to systematically evaluate the therapeutic effects of BSO on MAFLD and to explore the underlying molecular mechanisms with a particular focus on gut microbiota structure, intestinal barrier function, the intestinal LXRα/ABCA1 and FXR/FGF15 signaling pathways, hepatic metabolite profiles, and bile acid metabolism.

## 2 Materials and methods

### 2.1 Animals

Fifty-six specific pathogen-free (SPF) male C57BL/6J mice, aged 6–7 weeks and weighing 16–19 g, were purchased from the Guangdong Medical Laboratory Animal Center (No. SCXK (Guangdong) 2022-0002). The mice were housed in the SPF Laboratory Animal Center of Guangdong Pharmaceutical University [No. SYXK (Guangdong) 2022-0125] under controlled conditions, Temperature 22–26°C, Relative humidity 55–65%, and a 12 h light and dark cycle. All animal experiments were approved by the Institutional Animal Care and Use Committee of Guangdong Pharmaceutical University (No. gdpulacspf2022591) and were conducted in strict accordance with the Guidelines for Ethical Review of Laboratory Animal Welfare (GB/T 35892-2018).

### 2.2 Drugs and reagents

Bletilla striata oligosaccharides (BSO, sugar content ≥ 98.0%, Mn = 1091 Da, Lot No. JTSW240425-1) were used. The monosaccharide composition and molecular weight determination of BSO are presented in Supplementary Material 1. Metformin (purity ≥ 99%) was purchased from Shanghai Macklin Biochemical Technology Co., Ltd. (Lot No.: C16124116). High-fat diet (HFD, 60% kcal from fat) was purchased from Research Diets, Inc., United States (Cat. No.: D12492). Normal chow diet was purchased from Chengdu Hualanxu Biotechnology Co., Ltd. (Cat. No.: HLX1010001); The energy composition is provided in Supplementary Material 2.

Biochemical assay kits for triglycerides (TG), total cholesterol (TC), low-density lipoprotein cholesterol (LDL-C), high-density lipoprotein cholesterol (HDL-C), total bile acids (TBA), alanine aminotransferase (ALT) and aspartate aminotransferase (AST) were purchased from Nanjing Jiancheng Bioengineering Institute. Enzyme-linked immunosorbent assay (ELISA) kits for insulin, interleukin-1β (IL-1β), interleukin-10 (IL-10), tumor necrosis factor-α (TNF-α), lipopolysaccharide (LPS), diamine oxidase (DAO), and D-lactic acid (D-LA) were purchased from Jiangsu Meimian Industrial Co., Ltd. The BCA protein assay kit, Western blot blocking buffer, and enhanced chemiluminescence (ECL) substrate were purchased from Beyotime Biotechnology Co., Ltd. (Shanghai, China). RIPA lysis buffer, phosphatase inhibitors, and protease inhibitors were purchased from Dalian Meilun Biotechnology Co., Ltd. Polyvinylidene difluoride (PVDF) membranes were purchased from Merck Millipore. Antibodies against Occludin, Claudin-1, LXRα, ABCG8, CD14, TLR4, MYD88, p65-NF-κB, GAPDH, Horseradish peroxidase (HRP)-conjugated were purchased from Proteintech Group, ZO-1, FXR, FGF15, β-actin were purchased from Abcam, ABCA1 was purchased from Invitrogen, and p-p65-NF-κB was purchased from SAB.

### 2.3 Animal model establishment and drug administration

After 1 week of acclimatization, the mice were randomly divided into two groups, the normal control group (Control, *n* = 12) fed a normal chow diet, and the model group (*n* = 44) fed a high-fat diet. Body weight and food intake were recorded weekly during the modeling period. After 8 weeks, four mice were randomly selected from each group for body weight measurement and liver histopathological examination to confirm successful MAFLD model establishment (38).

The 40 successfully modeled mice were randomly divided into five groups (*n* = 8 per group): model group (Model), positive control group (Metformin, 50 mg/kg), BSO low-dose group (BSO-L, 150 mg/kg), BSO medium-dose group (BSO-M, 300 mg/kg), and BSO high-dose group (BSO-H, 600 mg/kg). The dosage of metformin was based on previous studies from our research group (38), and the dosages of BSO were determined based on previous reports (35). The Control and Model groups received an equal volume of normal saline by oral gavage, while the other groups received the corresponding drugs once daily for 12 consecutive weeks (38).

### 2.4 Glucose tolerance test

After 12 weeks of treatment, the mice were fasted for 12 h with free access to water, and fasting blood glucose levels were measured (0 min). Subsequently, glucose solution was administered by gavage at a dose of 2.0 g/kg body weight. Blood glucose levels were measured at 15, 30, 60, 90, and 120 min after glucose administration using tail vein blood. The area under the curve (AUC) was calculated (38).

### 2.5 Histopathological staining

#### 2.5.1 H&E staining of liver and colon tissues

Tissues were fixed in 4% paraformaldehyde for 24 h, rinsed under running water for 30 min, dehydrated through a graded ethanol series, cleared in xylene, and embedded in paraffin using a paraffin embedding station (HistoCore Arcadia, Leica, Wetzlar, Germany). Sections of 4 μm thickness were prepared using a rotary microtome (RM2255, Leica, Wetzlar, Germany). After deparaffinization and rehydration, sections were stained with hematoxylin for 5 min, differentiated with 1% hydrochloric acid in ethanol, and counterstained with eosin for 2 min. After dehydration and clearing, sections were mounted with neutral balsam. Histopathological morphology of the liver and colon was observed under a light microscope (BX53, Olympus, Tokyo, Japan).

#### 2.5.2 Oil Red O staining of liver tissues

Fresh liver tissues were embedded in OCT compound, and 6 μm cryosections were prepared using a cryostat (CM1950, Leica, Wetzlar, Germany). Sections were fixed in 4% paraformaldehyde for 2 min, immersed in 60% isopropanol for 20 s, and stained with Oil Red O solution for 3–5 min. After differentiation with 1% hydrochloric acid in ethanol, sections were rinsed under running water for 10 min and mounted with glycerol gelatin. Images were captured under a light microscope (BX53, Olympus, Tokyo, Japan).

### 2.6 Western blot

Liver or colon tissues (50 mg) were homogenized in 500 μL of RIPA lysis buffer containing phosphatase inhibitors and protease inhibitors using a tissue grinder (TissueLyser II, Qiagen, Hilden, Germany) at 50 Hz for 10 min. The homogenates were centrifuged at 12,000 rpm for 15 min at 4°C using a centrifuge (Centrifuge 5418R, Eppendorf, Hamburg, Germany), and the supernatants were collected. Protein concentrations were determined using the BCA assay. Protein concentrations were adjusted to 5.0 μg/μL for liver tissues and 2.0 μg/μL for colon tissues, mixed with 5 × protein loading buffer, and denatured at 98°C for 10 min. Proteins were separated by SDS-PAGE using 10% separating gels and 5% stacking gels in an electrophoresis apparatus (Mini-PROTEAN Tetra system, Bio-Rad, Hercules, CA, United States), with 20 μg of total protein loaded per well. Electrophoresis was performed at 80 V until the bromophenol blue dye entered the separating gel, followed by 120 V until the dye reached the bottom of the gel. Proteins were transferred onto PVDF membranes using a wet transfer apparatus (Trans-Blot Turbo system, Bio-Rad, Hercules, CA, United States) at 250 mA for 75 min. Membranes were blocked with rapid blocking buffer for 10 min and washed three times with TBST. Membranes were incubated with primary antibodies overnight at 4°C, followed by three washes with TBST and incubation with HRP-conjugated secondary antibodies for 90 min at room temperature. The dilutions of primary antibodies are listed in Supplementary Material 3. After washing, protein bands were visualized using ECL substrate on a chemiluminescence imaging system (ChemiDoc MP, Bio-Rad, Hercules, CA, United States). Band intensities were quantified using Image J software (version 1.53, National Institutes of Health, Bethesda, MD, United States), and relative protein expression levels were normalized to GAPDH.

### 2.7 qRT-PCR analysis

Total RNA was extracted from tissues using RNAiso Plus reagent (Takara Bio, Japan). RNA concentration and purity were measured using a NanoDrop 2000 spectrophotometer (Thermo Fisher Scientific, United States). A total of 1000 ng of RNA was reverse-transcribed into cDNA using the PrimeScript RT reagent Kit (Takara, Cat# RR037A) on a T100 Thermal Cycler (Bio-Rad, United States). The resulting cDNA was stored at –20°C.

qRT-PCR was performed using TB Green Premix Ex Taq II (Tli RNaseH Plus) (Takara, Cat# RR82A) on a QuantStudio 5 Real-Time PCR System (Thermo Fisher Scientific). The 10 μL reaction mixture contained: 2 μL cDNA, 0.75 μL forward primer and 1 μL reverse primer (both at 10 μmol/L), 6 μL PCR reaction mix, and 2.5 μL nuclease free water. The PCR protocol was as follows: denaturation at 95°C for 30 s; annealing at 60°C for 30 s for 30 cycles; and extension at 72°C for 30 s. The relative gene expression was analyzed using the 2^–Δ^ ^Δ^ *^Ct^* method, with GAPDH serving as the internal reference gene. Details of the gene primers used are provided in Supplementary Material 4.

### 2.8 16 S rRNA gene sequencing and microbiota analysis

Cecal contents were collected from six mice in each of the Control, Model, and BSO-H groups. Total DNA was extracted using a DNA extraction kit (QIAamp PowerFecal Pro DNA Kit, Qiagen, Hilden, Germany), and PCR amplification was performed using a thermal cycler (Veriti 96-well thermal cycler, Applied Biosystems, Waltham, MA, United States) with barcoded primers targeting the V3–V4 region of the 16S rRNA gene (515F: 5′-GTGCCAGCMGCCGCGGTAA-3′;806R: 5′-GGACTACHVGGGTWTCTAAT-3′). PCR products were purified, and libraries were constructed for high-throughput sequencing on the Illumina NovaSeq 6000 platform (Illumina, San Diego, CA, United States). After quality filtering and chimera removal, sequences were clustered into amplicon sequence variants (ASVs) at 97% similarity. Alpha diversity and beta diversity analyses were performed using QIIME2 software, and taxonomic annotation was conducted based on the Silva database (39).

### 2.9 Untargeted hepatic metabolomics analysis

Liver tissue (20 mg) was homogenized with steel beads and 400 μL of 70% methanol in water containing internal standards using a ball mill (MM400 mixer mill, Retsch, Haan, Germany) at 30 Hz for 20 s. The homogenate was centrifuged at 12,000 rpm for 10 min at 4°C, and 300 μL of supernatant was collected and incubated at –20°C for 30 min. After a second centrifugation, 200 μL of supernatant was transferred for LC-MS/MS analysis. Chromatographic separation was performed on a Waters ACQUITY Premier HSS T3 column (1.8 μm, 2.1 mm × 100 mm) using mobile phase A (0.1% formic acid in water) and mobile phase B (0.1% formic acid in acetonitrile). The column temperature was maintained at 40°C, and the flow rate was 0.4 mL/min. Mass spectrometry was performed using an electrospray ionization (ESI) source in both positive and negative ion modes, with a mass scan range of 75–1,000 Da. Raw data were converted to mzML format using ProteoWizard, and peak picking, alignment, and retention time correction were performed using XCMS. Metabolite identification was based on an in-house standard database and public databases.

### 2.10 Targeted bile acid metabolomics analysis of cecal contents

Cecal content (20 mg) was mixed with 10 μL of internal standard working solution (1 μg/mL) and 500 μL of methanol, homogenized, and vortexed at 2,500 rpm for 10 min. After incubation at –20°C for 10 min, the mixture was centrifuged at 12,000 rpm for 10 min at 4°C. The supernatant was processed through a protein precipitation plate prior to LC-MS/MS analysis. Chromatographic separation was performed on a Waters ACQUITY UPLC HSS T3 C18 column (1.8 μm, 100 mm × 2.1 mm, Waters, Milford, MA, United States) using mobile phase A (ultrapure water containing 0.01% acetic acid and 5 mmol/L ammonium acetate) and mobile phase B (acetonitrile containing 0.01% acetic acid). The flow rate was 0.35 mL/min, and the column temperature was 40°C. Mass spectrometry was performed using a QTRAP 6500+ mass spectrometer (Sciex, Framingham, MA, United States) equipped with an ESI source in negative ion mode with multiple reaction monitoring (MRM) for qualitative and quantitative analysis. Data processing was performed using MultiQuant software. Metabolites were filtered (missing rate ≤ 50% in any experimental group and overall missing rate ≤ 50%), and missing values were imputed using half of the minimum value. A total of 54 bile acids were retained for subsequent statistical analysis (35).

### 2.11 Statistical analysis

Statistical analyses were performed using GraphPad Prism 9.5 software. Quantitative data are presented as mean ± SD. Comparisons among multiple groups were performed using one-way analysis of variance (ANOVA), followed by Tukey’s *post-hoc* test. Comparisons between two groups were performed using independent samples *t*-test. *p* < 0.05 was considered statistically significant.

## 3 Results

### 3.1 Body weight changes and liver injury in MAFLD mice during model establishment

**FIGURE 1:**
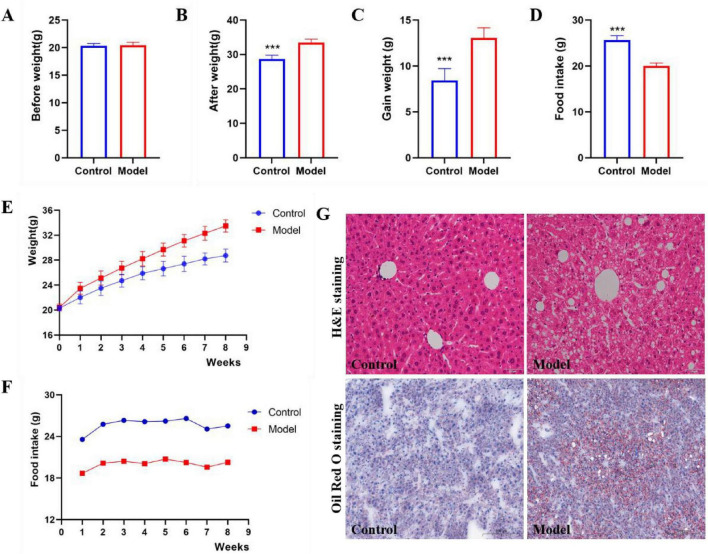
**(A)** Initial body weight of mice before dietary intervention. **(B)** Body weight after 8 weeks of feeding with normal chow diet or high-fat diet. **(C)** Body weight gain after 8 weeks of modeling. **(D)** Average weekly food intake per mouse during the modeling period. **(E)** Weekly body weight changes during the modeling period. **(F)** Weekly food intake changes during the modeling period. **(G)** Liver sections stained with H&E (50 μm) and Oil Red O (200 μm). All data are presented as mean ± standard deviation (SD). Comparisons between the control (*n* = 8) and model (*n* = 40) groups were performed using unpaired two-tailed Student’s t-test. Significance markers in the figure: *p < 0.05, **p < 0.01, ***p < 0.001. Error bars represent SD.

After 1 week of acclimatization, there was no significant difference in initial body weight between the Control group (*n* = 12) and the Model group (*n* = 44) (Figure 1A). Following 8 weeks of feeding with either a normal chow diet or a high-fat diet, body weight significantly differed between the two groups (*p* < 0.001) (Figures 1B,E). Compared with the Control group, the Model group exhibited a significant increase in body weight (*p* < 0.001) (Figure 1C). Food intake was significantly lower in the Model group than in the Control group (*p* < 0.001) (Figures 1D,F). A possible reason for this reduction is that the high-fat diet has a higher energy density than the normal chow diet, which may trigger compensatory regulatory mechanisms in the animals to limit oral intake. However, due to the markedly higher total energy content of the high-fat diet compared with the normal chow diet, the actual energy intake of the Model group remained significantly elevated, ultimately leading to substantial weight gain. H&E and Oil Red O staining revealed that the Control group displayed clear hepatic lobular architecture with well-arranged hepatic cords. In contrast, the Model group showed marked macrovesicular steatosis with diffuse lipid droplet accumulation and nuclei displaced to the periphery of hepatocytes (Figure 1G). These findings collectively indicate successful establishment of the MAFLD mouse model.

### 3.2 BSO alleviates body weight gain and insulin resistance in MAFLD mice

**FIGURE 2:**
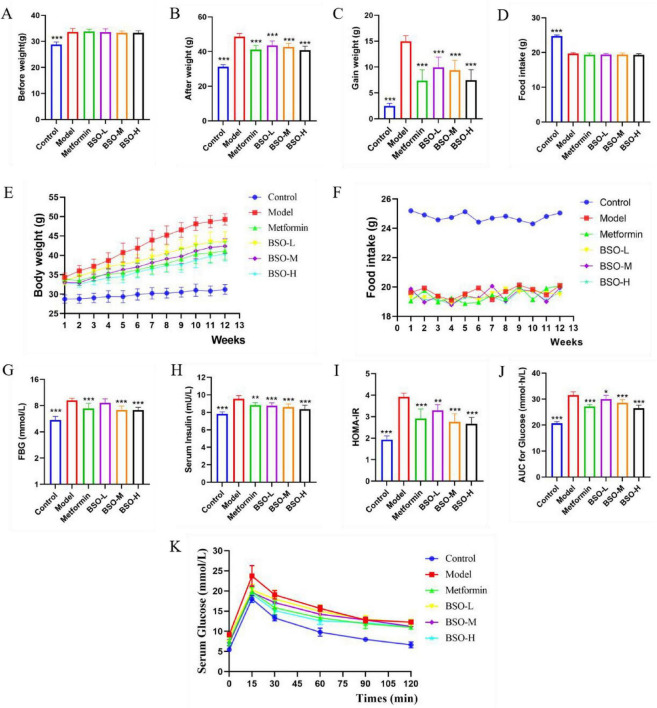
**(A)** Body weight before treatment. **(B)** Body weight after 12 weeks of treatment. **(C)** Body weight gain after the treatment. **(D)** Average weekly food intake per mouse. **(E)** Body weight changes over the 12 weeks treatment period. **(F)** Food intake changes over the 12 weeks treatment period. **(G)** FBG levels after treatment. **(H)** Serum insulin levels. **(I)** HOMA-IR index. **(J)** AUC of OGTT. **(K)** Blood glucose levels at different time points during OGTT. All data are presented as mean ± standard deviation (SD) from eight biologically independent mice per group (*n* = 8). One-way ANOVA with Tukey’s multiple-comparison test was used for statistical comparisons between groups. Significance markers in the figure: *p < 0.05, **p < 0.01, ***p < 0.001. Error bars represent SD.

After model establishment, the Model group mice were randomly divided into five subgroups, with no significant difference in body weight among the groups (*p* > 0.05) (Figure 2A). To evaluate the therapeutic effect of BSO on MAFLD, mice received metformin or BSO intervention for 12 weeks. Compared with the Control group, the Model group exhibited significantly increased body weight and body weight gain (*p* < 0.001). Compared with the Model group, the BSO-L, BSO-M, and BSO-H groups showed significantly reduced body weight and body weight gain (*p* < 0.001), with the BSO-H group demonstrating comparable efficacy to the metformin group (Figures 2B,C,E). No significant differences in food intake were observed among the groups (Figures 2D,F). This finding suggests that the weight-reducing effect of BSO is not mediated by appetite suppression, but rather through direct metabolic regulation. Fasting blood glucose, serum insulin levels, and OGTT results indicated that metformin or BSO treatment improved glucose tolerance and insulin resistance in HFD-induced mice. Compared with the Control group, the Model group exhibited significantly elevated fasting blood glucose, serum insulin levels, and HOMA-IR index (*p* < 0.001). Following BSO intervention, these parameters decreased in a dose-dependent manner, with the BSO-H group showing effects comparable to those of the metformin group (Figures 2G–K). Collectively, these findings demonstrate that BSO alleviates body weight gain and ameliorates glucose intolerance and insulin resistance in MAFLD mice. The underlying mechanisms may involve redox-dependent metabolic reprogramming, enhanced fatty acid oxidation, and attenuation of lipotoxicity in insulin-responsive tissues.

### 3.3 BSO ameliorates lipid metabolism disorders and liver injury in MAFLD mice

**FIGURE 3:**
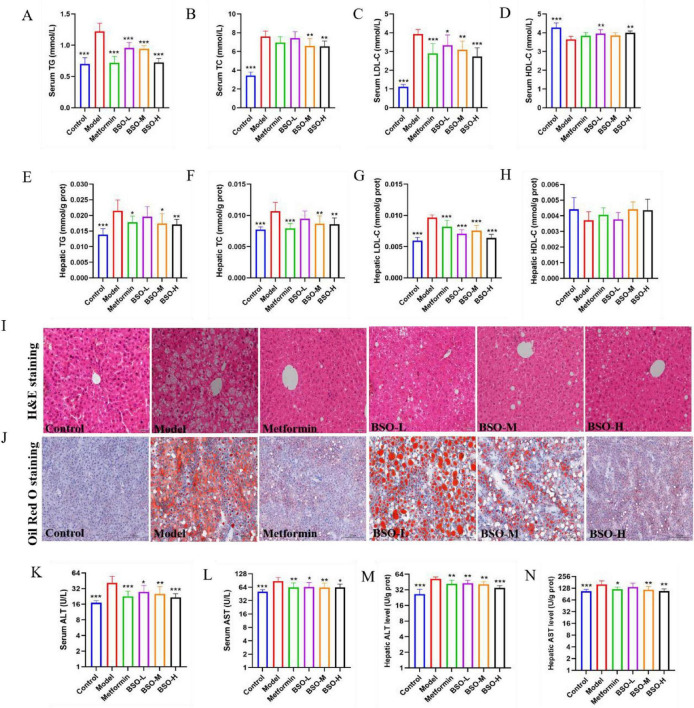
**(A)** Serum TG levels. **(B)** Serum TC levels. **(C)** Serum LDL-C levels. **(D)** Serum HDL-C levels. **(E)** Hepatic TG levels. **(F)** Hepatic TC levels. **(G)** Hepatic LDL-C levels. **(H)** Hepatic HDL-C levels. **(I)** Representative images of liver H&E staining (50 μm). **(J)** Representative images of liver Oil Red O staining (200 μm). **(K)** Serum ALT activity. **(L)** Serum AST activity. **(M)** Hepatic ALT activity. **(N)** Hepatic AST activity. All data are presented as mean ± standard deviation (SD) from eight biologically independent mice per group (*n* = 8). One-way ANOVA with Tukey’s multiple-comparison test was used for statistical comparisons between groups. Significance markers in the figure: *p < 0.05, **p < 0.01, ***p < 0.001. Error bars represent SD.

To evaluate the effects of BSO on lipid metabolism and liver injury in MAFLD mice, we measured serum and hepatic lipid levels, liver function biomarkers, and performed liver histopathological staining. Compared with the Control group, the Model group exhibited significantly elevated levels of TG, TC, and LDL-C in both serum and liver tissue (*p* < 0.001), while serum HDL-C levels showed a decreasing trend without statistical significance (*p* > 0.05). Following BSO or metformin intervention, these lipid parameters were significantly improved, as evidenced by reduced TG, TC, and LDL-C levels and increased HDL-C levels (Figures 3A–H).

H&E and Oil Red O staining revealed normal hepatic morphology in the Control group. Compared with the Model group at the end of the 8-week modeling period, the untreated Model group after an additional 12 weeks of continuous HFD feeding exhibited more severe hepatic steatosis, characterized by denser lipid vacuoles on H&E staining and more extensive red-stained lipid droplet areas on Oil Red O staining. Following BSO or metformin treatment, hepatocyte steatosis was markedly alleviated, lipid droplet accumulation was significantly reduced, and hepatic histopathological morphology was substantially improved (Figures 3I,J). Excessive hepatic lipid accumulation can lead to hepatocyte injury, manifested as abnormally elevated ALT and AST activities in serum and liver tissue. Liver function analysis showed that compared with the Control group, ALT and AST activities in both serum and liver tissue were significantly increased in the Model group (*p* < 0.001). Following BSO or metformin intervention, these parameters were significantly decreased (*p* < 0.01) (Figures 3K–N). In brief, these results demonstrate that BSO treatment alleviates HFD-induced hepatic lipid accumulation and lipotoxic injury.

### 3.4 BSO mitigated intestinal barrier injury in MAFLD mice

The interplay between the liver and gut microbiota, along with the transmission of metabolites via the portal vein, underscores the critical role of the gut-liver axis in the pathogenesis, progression, and treatment of MAFLD (11). To evaluate the effects of BSO on intestinal barrier function in MAFLD mice, we performed histopathological assessment of colon tissues and measured serum intestinal barrier markers and tight junction protein expression.

**FIGURE 4:**
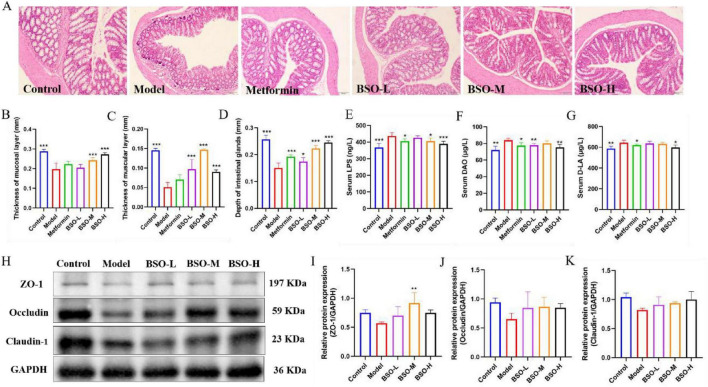
**(A)** Representative images of colon H&E staining (100 μm). **(B)** Quantitative analysis of colonic mucosal layer thickness. **(C)** Quantitative analysis of colonic muscular layer thickness. **(D)** Quantitative analysis of colonic crypt depth. **(E)** Serum LPS levels. **(F)** Serum DAO levels. **(G)** Serum D-LA levels. **(H)** Western blot bands showing colonic expression of ZO-1, Occludin, and Claudin-1. **(I)** Relative protein expression of ZO-1. **(J)** Relative protein expression of Occludin. **(K)** Relative protein expression of Claudin-1. All data are presented as mean ± standard deviation (SD). One-way ANOVA with Tukey’s multiple-comparison test was used for statistical comparisons between groups. Significance markers in the figure: *p < 0.05, **p < 0.01, ***p < 0.001. Error bars represent SD. Sample sizes (*n*) represent biologically independent mice per group: *n* = 8 for **(E–G)**, *n* = 6 for **(B–D)**, *n* = 3 for **(I–K)**.

H&E staining revealed intact colonic mucosal structure and regular crypt arrangement in the Control group. Compared with the Control group, the Model group exhibited irregular colonic crypt structure, with significantly reduced crypt depth, muscle layer thickness, and mucosal layer thickness (*p* < 0.001). Following BSO or metformin treatment, crypt depth, muscle layer thickness, and mucosal layer thickness were significantly improved (Figures 4A–D). Serum intestinal barrier marker analysis showed that serum levels of LPS, DAO, and D-LA were significantly elevated in the Model group (*p* < 0.01). Following BSO intervention, serum LPS, DAO, and D-LA levels were reduced in a dose-dependent manner (Figures 4E–G). Western blot analysis revealed that the expression of colonic tight junction proteins ZO-1, Occludin and Claudin-1 was significantly downregulated in the Model group, whereas BSO treatment significantly restored their expression levels (Figures 4H–K). In summary, these findings indicate that BSO alleviates intestinal mucosal barrier injury and restores intestinal barrier function in MAFLD mice.

### 3.5 BSO activates the intestinal LXRα/ABCA1 pathway and suppresses hepatic TLR4/NF-κB inflammatory signaling

**FIGURE 5:**
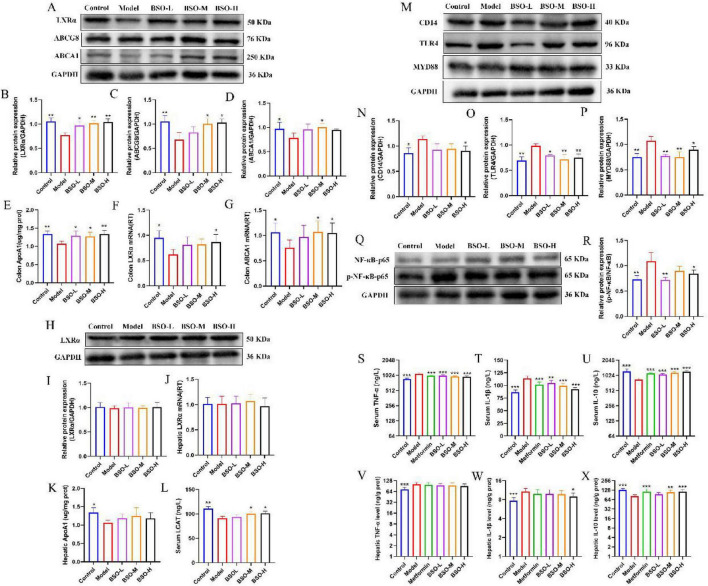
**(A)** Western blot bands of colonic LXRα, ABCA1, and ABCG8. **(B)** Relative protein expression of colonic LXRα. **(C)** Relative protein expression of colonic ABCA1. **(D)** Relative protein expression of colonic ABCG8. **(E)** Colonic ApoA1 levels. **(F)** Relative gene expression of colonic LXRα. **(G)** Relative gene expression of colonic ABCA1. **(H)** Western blot bands of hepatic LXRα. **(I)** Relative protein expression of hepatic LXRα. **(J)** Relative gene expression of hepatic LXRα. **(K)** Hepatic ApoA1 levels. **(L)** Serum LCAT levels. **(M)** Western blot bands of hepatic CD14, TLR4, and MYD88. **(N)** Relative protein expression of hepatic CD14. **(O)** Relative protein expression of hepatic TLR4. **(P)** Relative protein expression of hepatic MYD88. **(Q)** Western blot bands of hepatic p-NF-κB and NF-κB. **(R)** Ratio of p-NF-κB to NF-κB in the liver. **(S)** Serum TNF-α levels. **(T)** Serum IL-1β levels. **(U)** Serum IL-10 levels. **(V)** Hepatic TNF-α levels. **(W)** Hepatic IL-1β levels. **(X)** Hepatic IL-10 levels. All data are presented as mean ± standard deviation (SD). One-way ANOVA with Tukey’s multiple-comparison test was used for statistical comparisons between groups. Significance markers in the figure: *p < 0.05, **p < 0.01, ***p < 0.001. Error bars represent SD. Sample sizes (*n*) represent biologically independent mice per group: *n* = 8 for **(E,K–L,S–X)**, *n* = 6 for **(F,G,J)**, *n* = 3 for **(B–D,I,N–P,R)**.

To investigate the regulatory mechanism of BSO on lipid metabolism and inflammatory responses in the gut-liver axis, we examined key protein and gene expression in the colon and liver. In the colon, LXRα and ABCA1 (protein and mRNA), ABCG8 protein, and ApoA1 levels were significantly downregulated in the Model group (*p* < 0.05). Following BSO intervention, these parameters were significantly restored (Figures 5A–G). Notably, no significant differences were observed in hepatic LXRα mRNA or ApoA1 protein among groups, suggesting tissue-specific regulation of intestinal LXRα by BSO (Figures 5H–K). Serum LCAT levels were significantly reduced in the Model group (*p* < 0.01) and were significantly restored following BSO intervention (Figure 5L).

Hepatic inflammatory pathway analysis showed that CD14, TLR4, MYD88, and the p-NF-κB/NF-κB ratio were significantly elevated in the Model group (*p* < 0.05) and reduced by BSO (Figures 5M–P). ELISA results demonstrated that, compared with the Control group, the Model group had significantly higher TNF-α and IL-1β, and lower IL-10 in both serum and liver (*p* < 0.001). BSO intervention reversed these changes (Figures 5Q–X). These findings indicate that BSO activates the intestinal LXRα/ABCA1 pathway, thereby suppressing the hepatic LPS/TLR4/NF-κB inflammatory signaling cascade and ameliorating MAFLD-associated inflammatory responses.

### 3.6 BSO remodels the gut microbiota structure in MAFLD mice

**FIGURE 6:**
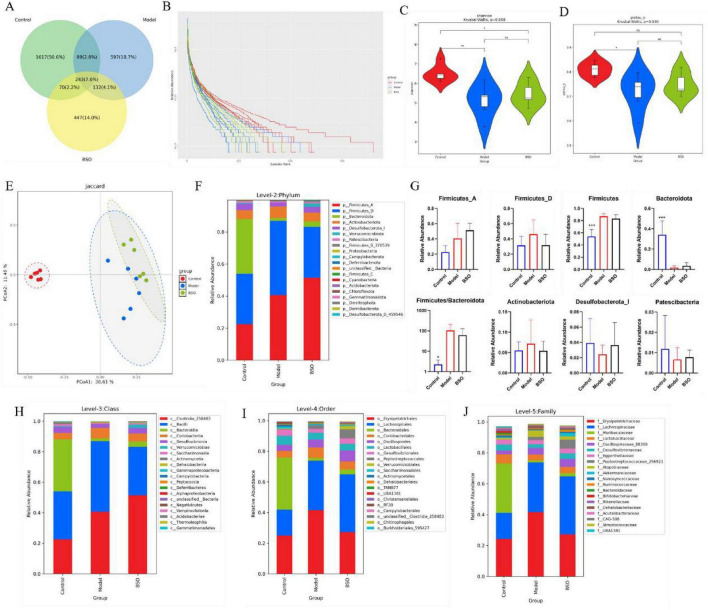
**(A)** Venn diagram showing ASV distribution among groups. **(B)** Rank-Abundance curves. **(C)** Shannon index. **(D)** Pielou evenness index. **(E)** PCoA plot. **(F)** Relative abundance of gut microbiota at the phylum level. **(G)** Comparison of differential phyla abundance. **(H)** Relative abundance of gut microbiota at the class level. **(I)** Relative abundance of gut microbiota at the order level. **(J)** Relative abundance of gut microbiota at the family level. All data are presented as mean ± standard deviation (SD) from eight biologically independent mice per group (*n* = 6). One-way ANOVA with Tukey’s multiple-comparison test was used for statistical comparisons between groups. Significance markers in the figure: *p < 0.05, **p < 0.01, ***p < 0.001. Error bars represent SD.

To further evaluate the effects of BSO on the gut microbiota, we analyzed cecal content samples using 16S rRNA sequencing. Venn diagrams illustrated the distribution of ASVs among the groups. A total of 243 ASVs were shared across the three groups. The Control group contained 1,617 unique ASVs, the Model group contained 597 unique ASVs, and the BSO group contained 447 unique ASVs (Figure 6A). Rank-Abundance curves showed that the Control group exhibited the widest horizontal span and the flattest vertical profile, indicating the highest species richness and the most even distribution of gut microbiota. In contrast, the Model and BSO groups displayed narrower horizontal spans and steeper curves, suggesting that HFD reduced both microbial richness and evenness. Following BSO intervention, the curve span increased slightly, indicating a partial improvement in microbial structure (Figure 6B). Alpha diversity analysis revealed that compared with the Control group, the Shannon index and Pielou evenness index were significantly reduced in the Model group (*p* < 0.05). Following BSO intervention, both the Shannon index and Pielou evenness index were significantly restored (Figures 6C,D). Principal coordinate analysis (PCoA) demonstrated a clear separation in microbial community structure between the Control and Model groups, while a slight separation was observed between the BSO and Model groups, suggesting that BSO intervention partially reversed HFD-induced alterations in microbial structure (Figure 6E). Species composition analysis at the phylum level showed that the relative abundances of Firmicutes_D, Firmicutes_A, and Actinobacteriota were increased in the Model group, whereas those of Bacteroidota, Desulfobacterota_I, and Patescibacteria were decreased. Following BSO intervention, the abundances of these phyla were restored to varying degrees (Figures 6F,G). Compared with the Control group, the Firmicutes/Bacteroidetes ratio was significantly elevated in the Model group (*p* < 0.05), and showed a decreasing trend following BSO intervention (Figure 6G). At the family level, BSO intervention further increased the relative abundances of Lachnospiraceae and Oscillospiraceae_88309, both belonging to the Firmicutes_A phylum (Figures 6H–J). These bacteria are well-known butyrate-producing genera. Butyrate, a short-chain fatty acid, exerts anti-inflammatory effects, enhances intestinal barrier function, and regulates energy metabolism. Thus, BSO-mediated enrichment of these beneficial bacteria may represent a key mechanism underlying its therapeutic effects on MAFLD. Collectively, these results indicate that HFD induced significant alterations in the composition and abundance of the gut microbiota in MAFLD mice, while BSO intervention effectively remodeled the gut microbiota structure.

### 3.7 BSO modulates hepatic metabolomic profiles in MAFLD mice

**FIGURE 7:**
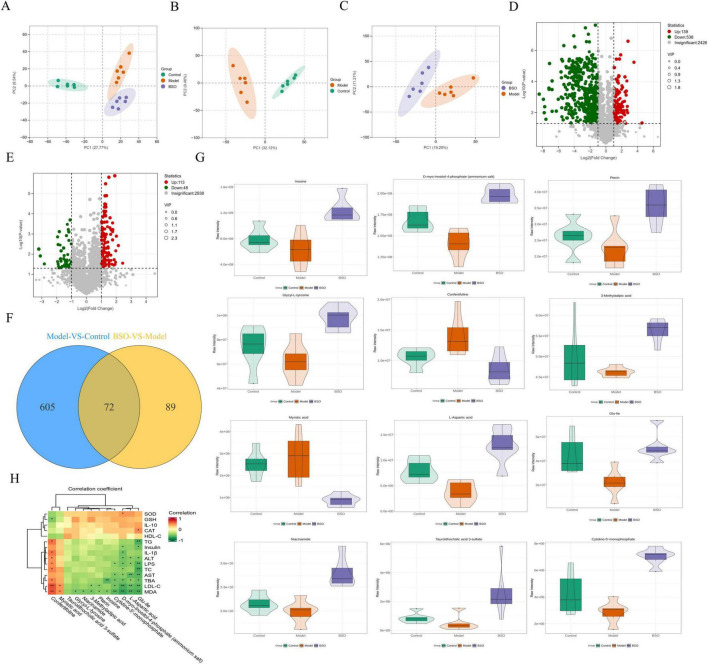
**(A)** PCA score plot. **(B)** OPLS-DA score plot between Control and Model groups. **(C)** OPLS-DA score plot between Model and BSO groups. **(D)** Volcano plot of differential metabolites between Control and Model groups. **(E)** Volcano plot of differential metabolites between Model and BSO groups. **(F)** Venn diagram of differential metabolites. **(G)** Relative expression levels of 12 key differential metabolites. **(H)** Heatmap of Pearson correlation analysis between 12 key differential metabolites and biochemical parameters. All data are presented as mean ± standard deviation (SD) from eight biologically independent mice per group (*n* = 6). One-way ANOVA with Tukey’s multiple-comparison test was used for statistical comparisons between groups. Error bars represent SD.

To investigate the effects of BSO on hepatic metabolites, we performed untargeted metabolomics analysis on liver samples from each group. Principal component analysis (PCA) revealed clear separation of metabolic profiles between the Control and Model groups, as well as between the Model and BSO groups (Figure 7A). Orthogonal partial least squares discriminant analysis (OPLS-DA) further validated the intergroup metabolic differences (Figures 7B,C). Differential metabolites were identified based on the following criteria: VIP > 1.0, FC > 2.0 or FC < 0.5, and *p* < 0.05. According to these criteria, 677 differential metabolites were identified between the Control and Model groups, of which 139 were upregulated and 538 were downregulated. Between the Model and BSO groups, 161 differential metabolites were identified, including 113 upregulated and 48 downregulated (Figures 7D,E). A total of 72 overlapping differential metabolites were identified between the two comparisons (Figure 7F). Further selection yielded 12 key differential metabolites closely associated with BSO treatment, including D-myo-Inositol-4-phosphate, Glycyl-L-tyrosine, Confertifoline, 3-Methyladipic acid, Pterin, Myristic acid, L-Aspartic acid, Glu-Ile, Inosine, Niacinamide, Taurolithocholic acid 3-sulfate, and Cytidine-5’-monophosphate (Figure 7G).

Pearson correlation analysis revealed that Confertifoline was significantly positively correlated with liver injury markers AST and ALT, lipid metabolism markers TG, TC, and LDL-C, total bile acids (TBA), lipopolysaccharide (LPS), and insulin levels, while it was significantly negatively correlated with anti-inflammatory cytokine IL-10. In contrast, D-myo-Inositol-4-phosphate and Glu-Ile showed positive correlations with protective markers and negative correlations with injury-related markers (Figure 7H). These results indicate that BSO intervention significantly alters the hepatic metabolic profile in MAFLD mice, with 12 key differential metabolites potentially involved in the therapeutic effects of BSO.

### 3.8 BSO activates the FXR/FGF15 signaling pathway and remodels bile acid metabolism

**FIGURE 8:**
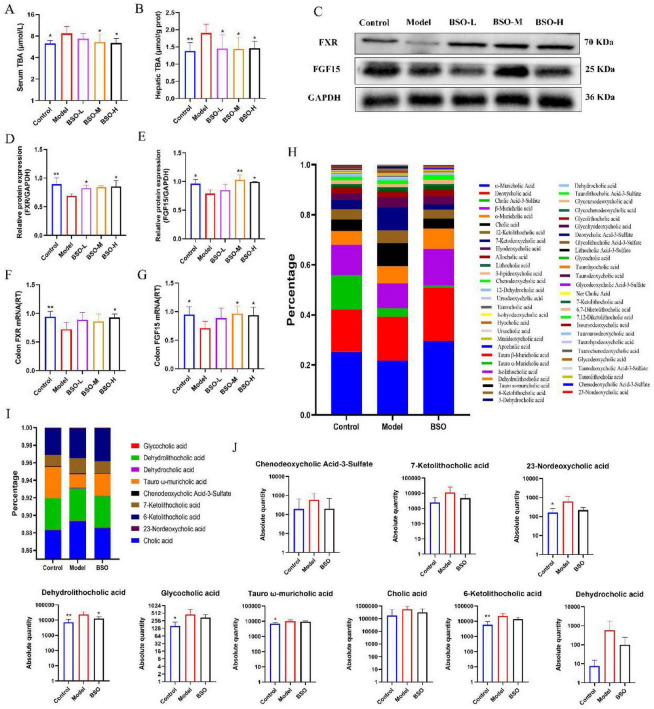
**(A)** Serum TBA levels. **(B)** Hepatic TBA levels. **(C)** Western blot bands of colonic FXR and FGF15 protein expression. **(D)** Relative protein expression of colonic FXR. **(E)** Relative protein expression of colonic FGF15. **(F)** Relative gene expression of colonic FXR. **(G)** Relative gene expression of colonic FGF15. **(H)** Composition and levels of the bile acid profile in cecal contents. **(I)** Composition chart showing the levels of nine significantly differential bile acids. **(J)** Comparison of nine significantly differential bile acids among groups. All data are presented as mean ± standard deviation (SD). One-way ANOVA with Tukey’s multiple-comparison test was used for statistical comparisons between groups. Error bars represent SD. Sample sizes (*n*) represent biologically independent mice per group: *n* = 8 for **(A,B)**, *n* = 6 for **(F–J)**, *n* = 3 for **(D,E)**.

To evaluate the effects of BSO on bile acid metabolism, we measured serum and hepatic TBA levels, colonic FXR and FGF15 mRNA and protein expression, and the bile acid profile in cecal contents. Compared with the Control group, serum and hepatic total bile acid levels were significantly elevated in the Model group (*p* < 0.05). Following BSO intervention, total bile acid levels were significantly reduced (*p* < 0.05) (Figures 8A,B). Western blot and qPCR analyses showed that colonic FXR and FGF15 protein and mRNA expression were significantly downregulated in the Model group (*p* < 0.05). Following BSO intervention, FXR and FGF15 protein expression were significantly restored (Figures 8C–G). Targeted bile acid metabolomics was performed to analyze the composition and levels of 54 bile acids in cecal contents. The results showed that HFD significantly altered the composition and levels of bile acids, whereas BSO intervention remodeled the bile acid profile (Figure 8H). Nine significantly differential bile acids were identified, including Tauro ω-muricholic acid, Glycocholic acid, Dehydrolithocholic acid, Dehydrocholic acid, Cholic acid, Chenodeoxycholic Acid-3-Sulfate, 7-Ketolithocholic acid, 6-Ketolithocholic acid, and 23-Nordeoxycholic acid (Figure 8I). Compared with the Control group, all nine bile acid levels were elevated in the Model group. Following BSO intervention, these bile acid levels were significantly reduced (Figure 8J). Notably, tauro-ω-muricholic acid is a natural antagonist of FXR; its reduction by BSO favors FXR activation. Cholic acid, a primary bile acid, is also reduced, reflecting global improvement in bile acid metabolism. These findings demonstrate that BSO activates the FXR/FGF15 signaling pathway and remodels the bile acid profile, thereby ameliorating MAFLD-associated bile acid metabolic disorders.

## 4 Discussion

The present study preliminarily reveals the mechanism by which BSO ameliorates HFD-induced MAFLD through multi-target regulation of the gut-liver axis. Specifically, BSO significantly improved the metabolic phenotype, liver injury, and inflammation in MAFLD mice; restored intestinal barrier integrity, activated the intestinal LXRα/ABCA1 pathway, and suppressed hepatic LPS/TLR4/NF-κB inflammatory signaling; remodeled the gut microbiota structure and modulated the hepatic metabolite profile; and concurrently activated the intestinal FXR/FGF15 signaling axis while correcting bile acid metabolic disorders. These findings provide experimental evidence supporting BSO as a novel therapeutic agent for MAFLD.

The pathogenesis of MAFLD involves complex interactions among insulin resistance, lipid metabolism disorders, and inflammatory responses (9, 10). In the present study, Model mice exhibited a typical MAFLD phenotype, including significantly increased body weight, glucose intolerance, insulin resistance, dyslipidemia, hepatic steatosis, and elevated transaminase levels. Following BSO intervention, these metabolic parameters were significantly improved, with the high-dose BSO group showing effects comparable to those of metformin. Notably, BSO did not affect food intake, indicating that its weight-reducing effect is not mediated by appetite suppression but may be associated with improved energy metabolism. This finding is consistent with a previous report showing that BSO regulates thermogenesis-related pathways (35).

Intestinal barrier dysfunction and subsequent LPS translocation are key drivers of hepatic inflammation in MAFLD (16, 22). In the present study, BSO significantly reduced serum levels of LPS, DAO, and D-LA, and upregulated the expression of colonic tight junction proteins ZO-1 and Occludin, indicating that BSO repairs HFD-induced intestinal barrier injury. Furthermore, BSO activated the colonic LXRα/ABCA1 signaling pathway. LXRα is a key nuclear receptor regulating cholesterol metabolism, and its activation promotes ABCA1-mediated cholesterol efflux in intestinal epithelial cells, leading to the formation of nascent high-density lipoprotein (20, 21). Gut-derived HDL possesses potent LPS-neutralizing capacity and can directly bind LPS in the portal circulation, thereby blocking its binding to TLR4 on hepatocytes (22, 25). Importantly, the capacity of HDL to neutralize LPS depends largely on its subfraction distribution. Previous studies have shown that large-sized HDL2 possesses the strongest LPS-binding capacity and significantly inhibits LPS-induced TNF-α release, whereas HDL3 exerts weaker effects, and preβ1-HDL lacks detectable anti-LPS activity (40). Recent studies have identified apolipoprotein M as a key mediator of the LPS-binding activity of HDL and have further demonstrated that HDL promotes LPS degradation via SR-B1, thereby suppressing IL-1β activation (41). Direct biophysical evidence has also shown that calcium enhances the adsorption of lipoproteins onto LPS-containing membranes, providing structural insights into the mechanism by which HDL neutralizes LPS (42). This subfraction dependent functionality has further been corroborated in serum assays from rats with high-fat diet-induced MAFLD following intervention with Atractylodes macrocephala extract (24). Colonic ABCA1 expression was significantly negatively correlated with serum LPS levels and hepatic TNF-α levels, supporting the involvement of gut-derived HDL in LPS clearance. Therefore, BSO may promote gut-derived HDL production by activating the intestinal LXRα/ABCA1 pathway, thereby eliminating translocated LPS at its source and suppressing the activation of the hepatic TLR4/NF-κB inflammatory pathway. However, this study only measured total HDL-C levels. Future studies should employ comprehensive HDL subfraction analysis to determine whether BSO preferentially induces HDL2 particles with potent anti-LPS activity, as suggested by the differential responses of HDL2 through HDL6 observed with other agents that target the gut-liver axis (24).

Gut microbiota dysbiosis is an initiating factor in MAFLD (13, 14). 16S rRNA sequencing results revealed that HFD significantly reduced gut microbial diversity and increased the Firmicutes/Bacteroidetes ratio. Although BSO intervention did not fully restore microbial richness, it significantly improved microbial evenness and promoted the proliferation of short-chain fatty acid (SCFA)-producing bacteria, including Lachnospiraceae and Oscillospiraceae. Lachnospiraceae are major butyrate producers in the gut. Butyrate serves as an energy source for intestinal epithelial cells, enhances intestinal barrier function, and exerts anti-inflammatory effects via G protein-coupled receptor activation (43, 44). Recent studies have shown that reduced Lachnospiraceae abundance in pediatric MAFLD patients correlates with elevated liver enzymes and insulin resistance, potentially through diminished flavone and flavonol biosynthesis pathways (45). Conversely, the enrichment of Lachnospiraceae observed in our study may contribute to improved metabolic outcomes via SCFA-dependent and -independent mechanisms. Oscillospiraceae is negatively associated with obesity and metabolic syndrome (46). Emerging evidence indicates that Oscillospiraceae abundance is lower in MAFLD patients than in healthy controls, and its enrichment may protect against hepatic steatosis progression (47). Although the specific metabolic functions of Oscillospiraceae remain incompletely characterized, its positive correlation with beneficial metabolic phenotypes highlights its potential as a gut health biomarker. BSO-mediated enrichment of these beneficial bacteria may synergistically enhance intestinal barrier function and anti-inflammatory effects through increased SCFA production. However, these findings are correlational rather than causal. Future studies employing fecal microbiota transplantation from BSO-treated donors into germ-free recipients, combined with targeted elimination of specific taxa, are warranted to establish causal relationships and elucidate the precise mechanisms by which these SCFA-producing bacteria contribute to the amelioration of MAFLD.

Untargeted hepatic metabolomics further revealed the regulatory effects of BSO on host metabolism. We identified 12 potential biomarkers associated with BSO treatment, among which confertifoline and myristic acid were significantly elevated in the MAFLD model and decreased following BSO intervention. Confertifoline is an alkaloid compound whose function remains unclear and requires further investigation. The present study found that confertifoline was significantly positively correlated with ALT, AST, TG, and LPS, suggesting its potential as a metabolic marker of MAFLD progression. Myristic acid is a saturated fatty acid, and elevated levels of myristic acid are closely associated with insulin resistance and hepatic steatosis (48). The reduction of myristic acid levels by BSO is consistent with its amelioration of insulin resistance and hepatic lipid accumulation. Furthermore, BSO elevated the levels of inosine and niacinamide. Inosine is an intermediate product of purine metabolism with antioxidant and anti-inflammatory properties, capable of protecting hepatocytes from damage (49). Niacinamide is a precursor of NAD^+^ and can improve mitochondrial function and insulin sensitivity by activating SIRT1 (50). These metabolite changes provide metabolic-level evidence for the multi-target effects of BSO.

Bile acid metabolic disorder is a prominent feature of MAFLD (28). In the present study, BSO significantly reduced serum and hepatic total bile acid levels and modulated the composition of nine key bile acids. Among these, BSO reduced the levels of dehydrolithocholic acid and dehydrocholic acid, both of which possess direct membrane-damaging effects and can induce apoptosis by damaging mitochondrial and cell membranes (51, 52). BSO also reduced the levels of the conjugated bile acids tauro ω-muricholic acid and glycocholic acid; excessive conjugated bile acids can disrupt the intestinal barrier and promote endotoxin translocation (53, 54).In summary, BSO can comprehensively regulate the activation of the FXR/FGF15 axis and reshape the composition of bile acids.

## 5 Conclusion

This study demonstrates that BSO effectively ameliorates HFD-induced MAFLD. The underlying mechanism is closely associated with regulation of the gut-liver axis, including remodeling of the gut microbiota, restoration of intestinal barrier integrity, activation of the LXRα/ABCA1 and FXR/FGF15 signaling pathways, and subsequent inhibition of the hepatic TLR4/NF-κB-mediated inflammatory response. These findings provide a theoretical basis for the application of BSO as a potential dietary strategy for MAFLD intervention. Future studies employing intestinal epithelial cell-specific LXRα knockout mouse models and comprehensive HDL subfraction analysis are warranted to further elucidate the tissue-specific regulatory mechanisms and lipid metabolism effects of BSO.

## Data Availability

The original contributions presented in the study are publicly available. This data can be found here: https://www.ncbi.nlm.nih.gov/bioproject/1481132 (accession number: PRJNA1481132).
