# Supplementary material for: Bletilla striata oligosaccharides alleviate high-fat diet-induced metabolic associated fatty liver in mice through modulation of gut microbiota and host metabolism
Source: Front Nutr. 2026 Jul 8;13:1852189. doi: 10.3389/fnut.2026.1852189 (PMC13388397; doi:10.3389/fnut.2026.1852189)
Supplement: Supplementary file 1 [file Table_1.docx]

**The Supplementary List**

**Supplementary 1:** Structural information of BSO.

**Supplementary 2:** Energy composition table of High-Fat diet and Normal chow diet.

**Supplementary 3:** Dilutions of primary antibodies.

**Supplementary 4:** Detailed information on animal ethics approval.

**Supplementary 1: Structural Information of BSO**

**1. Molecular Weight Determination of BSO by High-Performance Gel Permeation Chromatography**

The experiment was performed under the following conditions: mobile phase: 0.2 M NaCl solution; chromatographic column: BRT105-103-101 tandem gel column (8 × 300 mm); flow rate: 0.7 mL/min; column temperature: 40 °C; injection volume: 25 μL; detector: refractive index detector RID-20A. As shown in Figure S1-1 and Table S1-1 and Table S1-2, the Mn = 1091 Da.


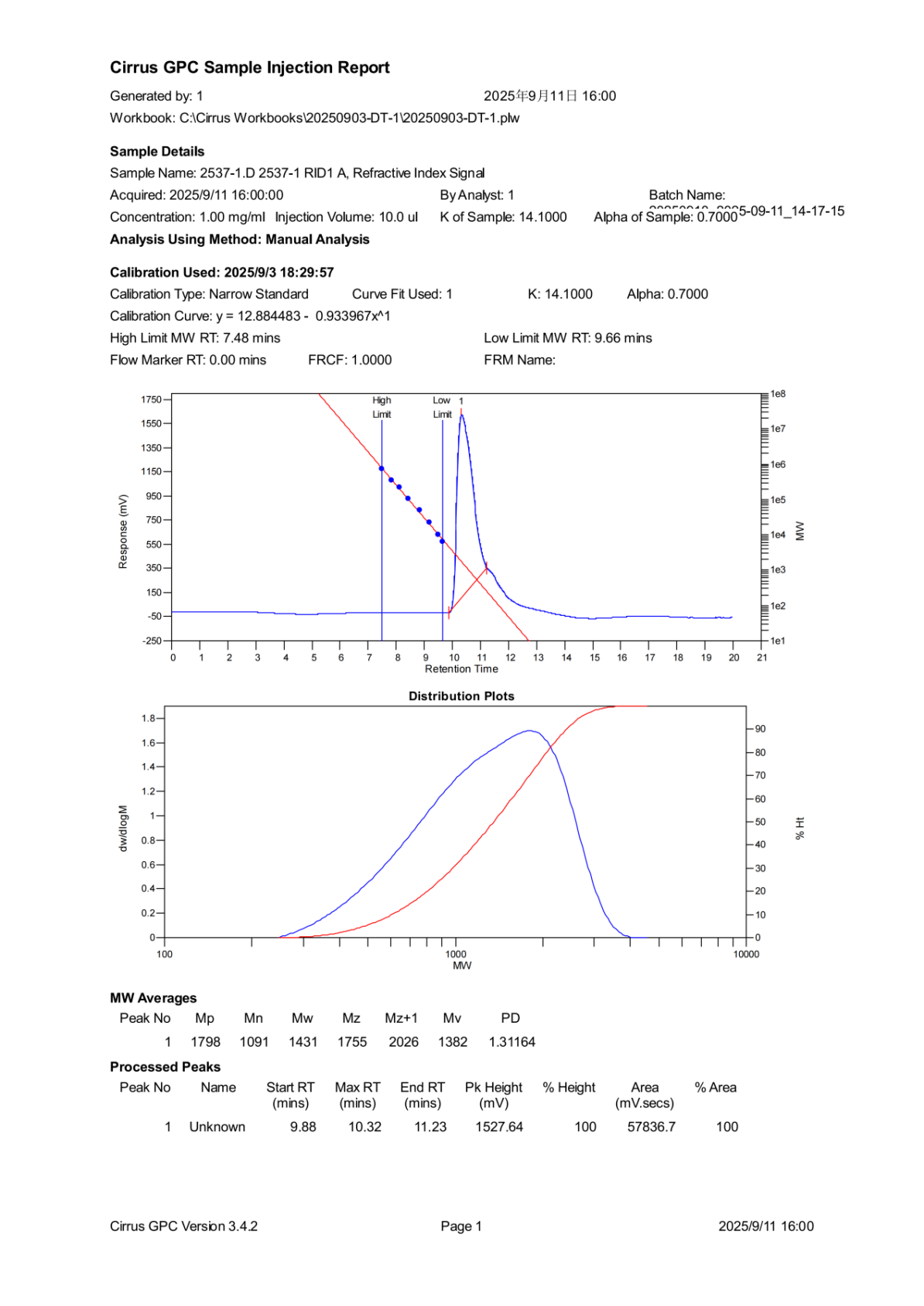


Figure S1-1 GPC chromatogram and molecular weight distribution of BSO

Table S1-1 Molecular weight averages of BSO

| Peak No | Mp | Mn | Mw | Mz | Mz+1 | Mv | PD |
| --- | --- | --- | --- | --- | --- | --- | --- |
| 1 | 1798 | 1091 | 1431 | 1755 | 2026 | 1382 | 1.31164 |

Table S1-2 Characteristic peaks of BSO

| Peak No | Start RT (mins） | Max RT (mins) | End RT (mins) | Pk Height (mV) | % Height | Area (mV.seces) | % Area |
| --- | --- | --- | --- | --- | --- | --- | --- |
| 1 | 9.88 | 10.32 | 11.23 | 1527.64 | 100 | 57836.7 | 100 |

**2. Analysis of Monosaccharide Composition by Ion Chromatography**

The experiment was performed under the following conditions: chromatographic column: Dionex Carbopac™ PA20 (3 mm × 150 mm); mobile phase: A: H₂O; B: 15 mM NaOH; C: 15 mM NaOH & 100 mM NaOAc; flow rate: 0.3 mL/min; injection volume: 25 µL; column temperature: 30 °C; the elution gradient was as follows: 0 min: A/B/C (99:1:0, V/V), 18 min: A/B/C (99:1:0, V/V), 20 min: A/B/C (75:25:0, V/V), 30 min: A/B/C (75:25:0, V/V), 30.1 min: A/B/C (20:0:80, V/V), 46 min: A/B/C (20:0:80, V/V), 46.1 min: A/B/C (0:100:0, V/V), 50 min: A/B/C (0:100:0, V/V), 50.1 min: A/B/C (99:1:0, V/V), 80 min: A/B/C (99:1:0, V/V). Detector: electrochemical detector. As shown in Figure S1-2 and Table S1-3, BSO is primarily composed of glucose and mannose, with a monosaccharide molar ratio of 0.312 and 0.662.


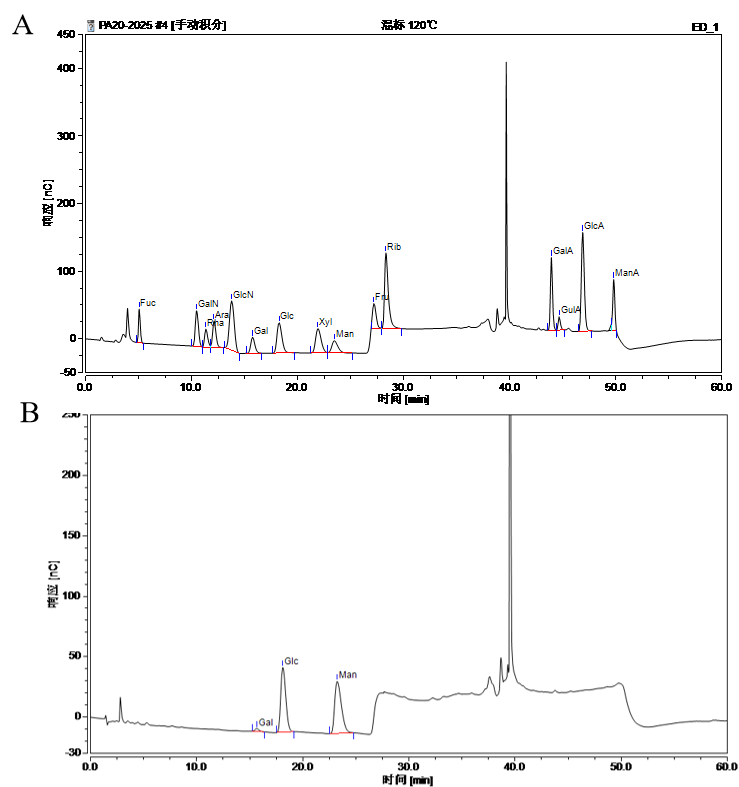


Figure S1-2 Ion chromatograms of the mixed standard (A) and BSO (B)

Table S1-3 Monosaccharide Composition of BSO

| Name | Peak Area | Retention Time | Molar Ratio | Content（µg/mg） |
| --- | --- | --- | --- | --- |
| Fuc | 0 | 5.06 | 0 | 0 |
| GalN | 0 | 10.47 | 0 | 0 |
| Rha | 0 | 11.35 | 0 | 0 |
| Ara | 0 | 12.11 | 0 | 0 |
| GlcN | 0 | 13.77 | 0 | 0 |
| Gal | 1.097 | 15.69 | 0.027 | 10.322 |
| Glc | 29.574 | 18.12 | 0.312 | 121.106 |
| Xyl | 0 | 21.91 | 0 | 0 |
| Man | 31.566 | 23.24 | 0.662 | 257.178 |
| Fru | 0 | 27.2 | 0 | 0 |
| Rib | 0 | 28.32 | 0 | 0 |
| GalA | 0 | 43.92 | 0 | 0 |
| GulA | 0 | 44.65 | 0 | 0 |
| GlcA | 0 | 46.87 | 0 | 0 |
| ManA | 0 | 49.8 | 0 | 0 |

1. **Structural Analysis of BSO by Fourier Transform Infrared Spectroscopy**

Preliminary structural analysis of BSO was performed using a Fourier transform infrared (FTIR) spectrometer. The measurement mode was transmission or attenuated total reflectance (ATR). The spectral scanning range was typically 4000–400 cm⁻¹, with a resolution of 4 cm⁻¹ and 32 scans accumulated to obtain spectra with a high signal-to-noise ratio. As shown in Figure S1-3, a broad and strong absorption band at 3347 cm⁻¹ corresponds to O–H stretching vibrations from intermolecular and intramolecular hydrogen bonding. The absorption bands at 2939 cm⁻¹ and 2905 cm⁻¹ are attributed to C–H stretching vibrations of methylene and methine groups in the sugar ring. A characteristic absorption at 1690 cm⁻¹ was observed, indicating the presence of C=O stretching vibrations. A series of absorption bands in the fingerprint region (1240–800 cm⁻¹), including peaks at 1243, 909, 856, and 801 cm⁻¹, are associated with sugar ring vibrations, glycosidic bonds (C–O–C), and anomeric carbon configuration, among which the absorption near 909 cm⁻¹ suggests the presence of β-type glycosidic linkages.


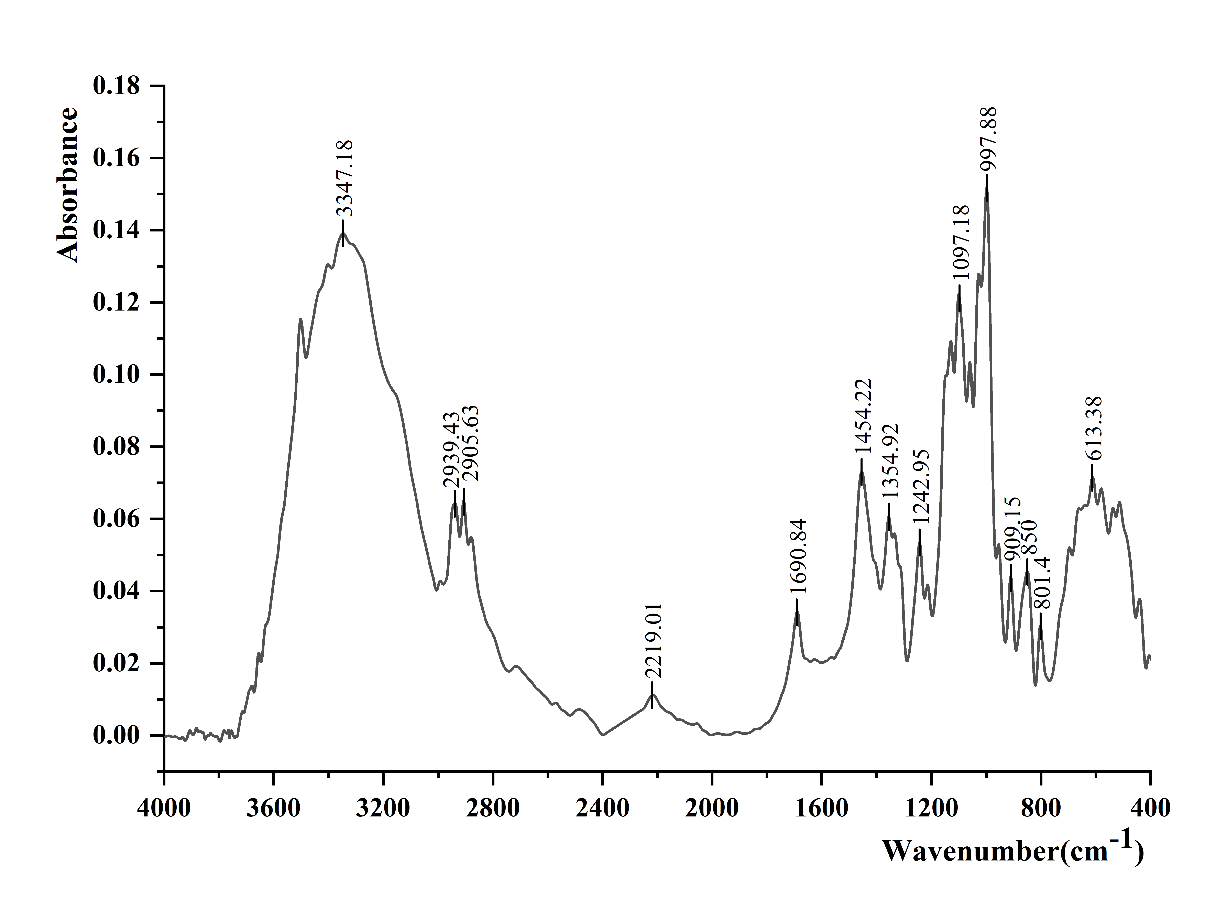


Figure S1-3 Fourier transform infrared (FTIR) spectrum of BSO

**Supplementary 2: Energy composition table of High-Fat diet and Normal chow diet**

| Diet | Component | gm% | kcal% | Energy (kcal/gm) |
| --- | --- | --- | --- | --- |
| Standard Diet | Protein | 20.61 | 23.79 | 3.46 |
|  | Fat | 5.8 | 15.06 |  |
|  | Carbohydrate | 52.99 | 61.15 |  |
| High-Fat Diet  (60% kcal fat, purified diet) | Protein | 26.2 | 20 | 5.24 |
|  | Fat | 34.9 | 60 |  |
|  | Carbohydrate | 26.3 | 20 |  |

**Supplementary 3: Dilutions of primary antibodies**

Table 3-4 Antibody information

| Antibody Name | Dilution Ratio | Manufacturer |
| --- | --- | --- |
| ZO-1 | 1:1000 | Abcam |
| Occludin | 1:1000 | Proteintech Group |
| Claudin-1 | 1:1000 | Proteintech Group |
| LXRα | 1:2000 | Proteintech Group |
| ABCA1 | 1:500 | Invitrogen |
| ABCG8 | 1:1000 | Proteintech Group |
| FXR | 1:5000 | Abcam |
| FGF15 | 1:2500 | Abcam |
| CD14 | 1:1000 | Proteintech Group |
| TLR4 | 1:1000 | Proteintech Group |
| MYD88 | 1:1000 | Proteintech Group |
| P65-NF-κB | 1:1000 | Proteintech Group |
| p-P65-NF-κB | 1:1000 | SAB |
| GAPDH | 1:5000 | Abcam |
| β-actin | 1:1000 | Proteintech Group |
| Goat Anti-Mouse | 1:5000 | Proteintech Group |

**Supplementary 4: Detailed information on animal ethics approval.**

**
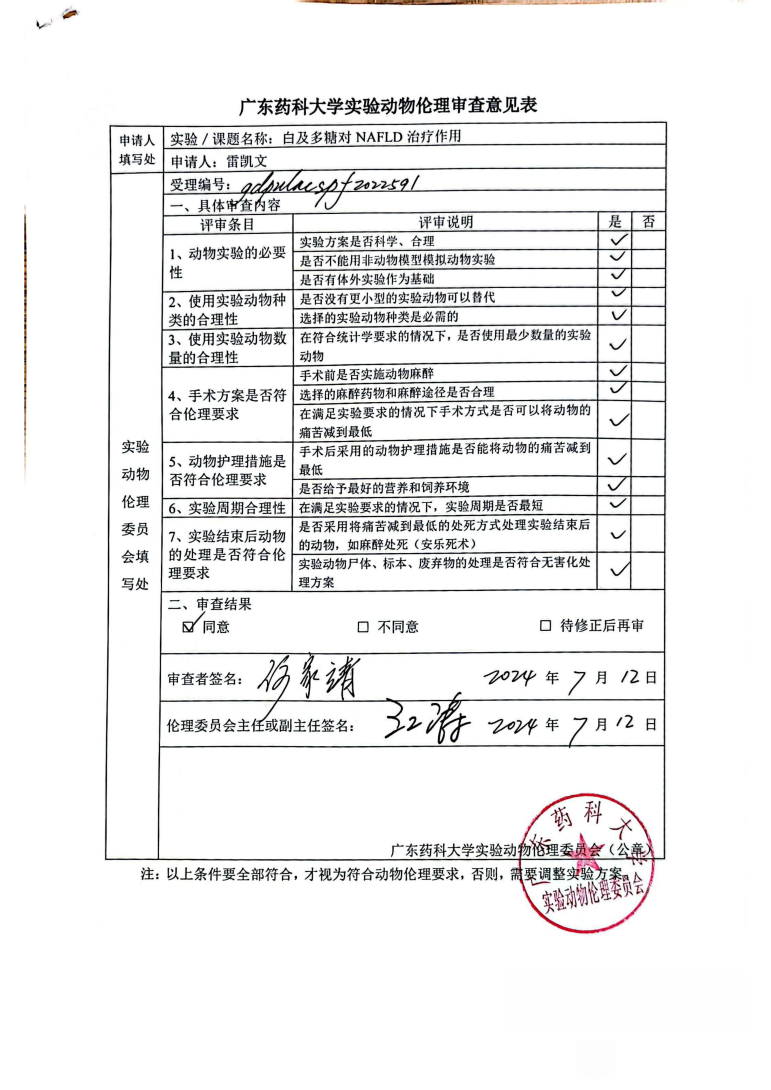
**
